# Supplementary material for: PLAbDab-nano: a database of camelid and shark nanobodies from patents and literature
Source: Nucleic Acids Res. 2024 Oct 10;53(D1):D535–42. doi: 10.1093/nar/gkae881 (PMC11701533; doi:10.1093/nar/gkae881)
Supplement: gkae881_Supplemental_File [file gkae881_supplemental_file.pdf]

Supplementary Information

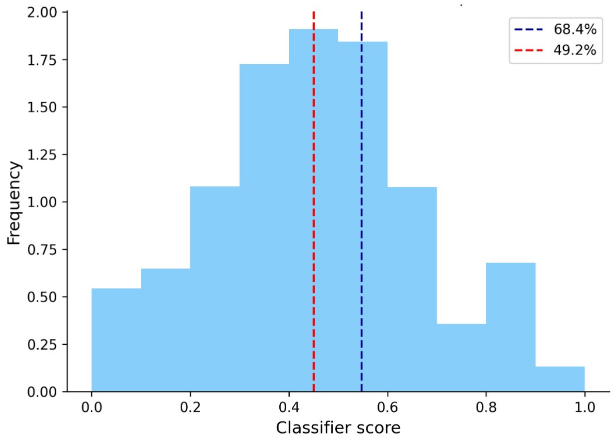

Figure S1. The distribution of classifier scores for sequences from patents and literature in the INDI database indicates that 68.4% of entries at our standard threshold of 0.55 (blue dashed line) and 49.2% at a relaxed threshold of 0.45 (red dashed line) would not pass our filtering methods, as they are classified as VH sequences.

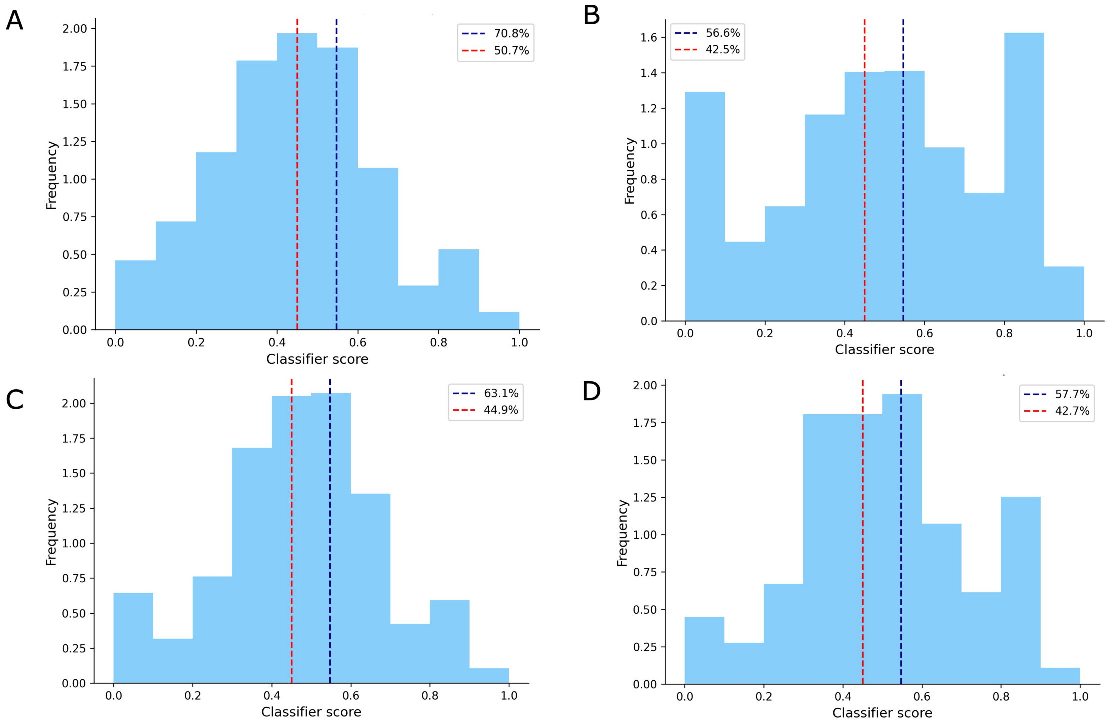

Figure S2. Distributions of classifier scores from INDI sequences, split by source into data from (**A**) patents, (**B**) GenBank, (**C**) structures, and (**D**) literature. Entries with scores to the left of the blue dashed lines (threshold = 0.55, calculated using Youden's J-statistic) and red dashed lines (where the threshold was relaxed to 0.45 to account for high VH and VHH sequence similarity) would not pass the filtering methods used to generate PLAbDab-nano, but are included in the INDI database. The values given in the figure legends next to the dashed lines indicate the percentage of sequences that would fail at that threshold.

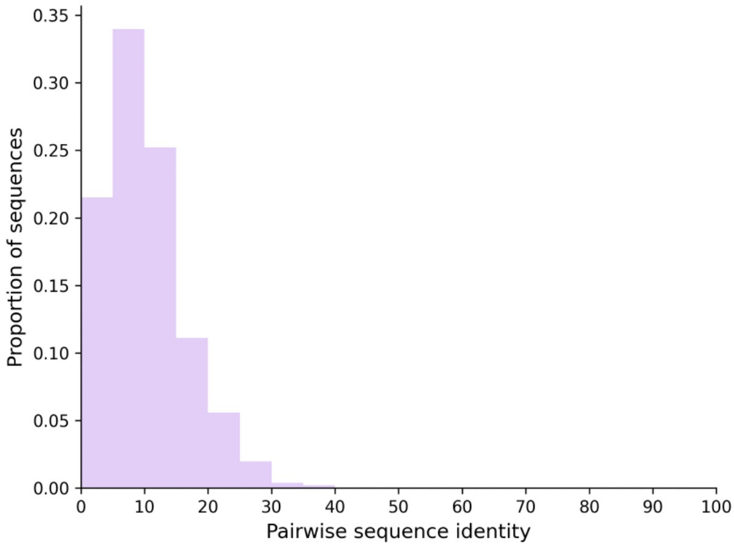

Figure S3. Low pairwise sequence identities (by percentage) for VHH CDR3 loops versus VNAR CDR3 loops indicate that the two types of nanobody occupy different regions of sequence space.

Table S1. VH sequences collected from GenBank and patents for the INDI database may be misclassified as nanobodies. Sources described in rows 1-7 do not contain nanobodies, row 8 describes an example where VH sequences are given and these are able to function as nanobodies.

	GenBank/patent ID	Number of sequences	Title
1	HE653038-HE653050, HE653053, HE653054, HE653057, HE653059-HE653070, HE653072, HE653078, HE653079, HE653083, HE653085, HE653086, HE653090, HE653091	35	Analysis of heavy and light chain sequences of conventional camelid antibodies from Camelus dromedarius and Camelus bactrianus species
2	AJ245151, AJ245168-AJ245173, AJ245178-AJ245198	25	Camel heavy-chain antibodies: diverse germline VHH and specific mechanisms enlarge the antigen-binding repertoire
3	US11530271	13	CD70 Combination Therapy
4	WO2013064701	68	Specific antibodies and their isolation with anti-idiotypic antibodies
5	US20220073604	105	IL-6 binding molecules
6	US20220177604	20	Anti-IgE antibodies
7	EP3061768	27	Camelid derived antigen binding polypeptides comprising a VH and VL chain
8	AF442089, AF442907-AF442929, AF442931-AF442934, AF442936, AF442938-AF442944, AF442946, AF442947, AF442949	38	Selection by phage display of llama conventional V(H) fragments with heavy chain antibody V(H)H properties
